# Supplementary material for: Relationship between Different Dimensions of Workplace Spirituality and Psychological Well-Being: Measuring Mediation Analysis through Conditional Process Modeling
Source: Int J Environ Res Public Health. 2022 Sep 7;19(18):11244. doi: 10.3390/ijerph191811244 (PMC9517529; doi:10.3390/ijerph191811244)
Supplement: Supplementary file 1 [file ijerph-19-11244-s001.zip › ijerph-1882122-supplementary.pdf]

```
FREQUENCIES VARIABLES=Gend MS Age Edu Exp Incm
  /ORDER=ANALYSIS.
```

Frequencies of respondents

Notes

Output Created		23-JUN-2022 13:41:56
Comments		
Input	Data	/Users/macbookpro/Documents/Data(Spritual).sav
	Active Dataset	DataSet1
	Filter	<none>
	Weight	<none>
	Split File	<none>
	N of Rows in Working Data File	873
Missing Value Handling	Definition of Missing	User-defined missing values are treated as missing.
	Cases Used	Statistics are based on all cases with valid data.
Syntax		FREQUENCIES VARIABLES=Gend MS Age Edu Exp Incm /ORDER=ANALYSIS.
Resources	Processor Time	00:00:00.00
	Elapsed Time	00:00:00.00

```
[DataSet1] /Users/macbookpro/Documents/Data(Spritual).sav
```

Statistics

		Gender	Marital Status	Age	Education	Experience	Income
N	Valid	873	873	873	873	873	873
	Missing	0	0	0	0	0	0

Frequency Table

Gender

		Frequency	Percent	Valid Percent	Cumulative Percent
Valid	Male	490	56.1	56.1	56.1
	Female	383	43.9	43.9	100.0
	Total	873	100.0	100.0	

Marital Status

		Frequency	Percent	Valid Percent	Cumulative Percent
Valid	Single	504	57.7	57.7	57.7
	Married	343	39.3	39.3	97.0

	Divorced	26	3.0	3.0	100.0
	Total	873	100.0	100.0	

Age

		Frequency	Percent	Valid Percent	Cumulative Percent
Valid	18-30	311	35.6	35.6	35.6
	30-40	196	22.5	22.5	58.1
	40-50	119	13.6	13.6	71.7
	50-60	138	15.8	15.8	87.5
	More than 60	109	12.5	12.5	100.0
	Total	873	100.0	100.0	

Education

		Frequency	Percent	Valid Percent	Cumulative Percent
Valid	High School	379	43.4	43.4	43.4
	Graduation	279	32.0	32.0	75.4
	Post Graduation	138	15.8	15.8	91.2
	Professional degree	77	8.8	8.8	100.0
	Total	873	100.0	100.0	

Experience

		Frequency	Percent	Valid Percent	Cumulative Percent
Valid	1-5	232	26.6	26.6	26.6
	5-10	263	30.1	30.1	56.7
	10-15	122	14.0	14.0	70.7

	15-20	117	13.4	13.4	84.1
	More than 20	139	15.9	15.9	100.0
	Total	873	100.0	100.0	

Income

		Frequency	Percent	Valid Percent	Cumulative Percent
Valid	10-30	150	17.2	17.2	17.2
	30-50	386	44.2	44.2	61.4
	50-70	179	20.5	20.5	81.9
	70-90	97	11.1	11.1	93.0
	More than 90	61	7.0	7.0	100.0
	Total	873	100.0	100.0	

```
DESCRIPTIVES VARIABLES=Psy_welbeing Work_Spritlty Compassion Relat_with_work Spritual_Orient
   Organz_Value Alingnm_Person_val Job_Stress Envonm_Mastery Persnal_Growth
  /STATISTICS=MEAN STDDEV KURTOSIS SKEWNESS.
```

Descriptive Analysis

Notes

Output Created		23-JUN-2022 13:43:07
Comments		
Input	Data	/Users/macbookpro/Documents/Data(Spritual).sav
	Active Dataset	DataSet1
	Filter	<none>
	Weight	<none>
	Split File	<none>
	N of Rows in Working Data File	873
Missing Value Handling	Definition of Missing	User defined missing values are treated as missing.
	Cases Used	All non-missing data are used.

Syntax		DESCRIPTIVES VARIABLES=Psy_welbeing Work_Spritlty Compassion Relat_with_work Spritual_Orient Organz_Value Alingnm_Person_val Job_Stress Envonm_Mastery Persnal_Growth /STATISTICS=MEAN STDDEV KURTOSIS SKEWNESS.
Resources	Processor Time	00:00:00.00
	Elapsed Time	00:00:00.00

Descriptive Statistics

	N	Mean	Std. Deviation	Skewness		Kurtosis	
	Statistic	Statistic	Statistic	Statistic	Std. Error	Statistic	Std. Error
Psychological Wellbeing	873	3.7892	1.08324	-.892	.083	.263	.165
Workplace Sprituality	873	3.7640	1.07909	-.821	.083	.184	.165
Compassion	873	3.9290	1.10856	-.953	.083	.313	.165
Relationship with others at work	873	3.8396	1.06096	-.930	.083	.515	.165
Spritual Orientation	873	3.7881	1.06648	-.804	.083	.274	.165
Organizational values	873	3.8202	1.04857	-.934	.083	.583	.165
Alignment of Personal values	873	3.9164	1.10508	-.943	.083	.313	.165
Job Stress	873	3.8625	1.07169	-.935	.083	.472	.165
Environmental Mastery	873	3.9278	1.10796	-.952	.083	.315	.165

Personal Growth	873	3.9003	1.09331	-.942	.083	.373	.165
Valid N (listwise)	873						

```
RELIABILITY
  /VARIABLES=Psy_welbeing Work_Spritlty Compassion Relat_with_work Spritual_Orient Organz_Value
    Alingnm_Person_val Job_Stress Envonm_Mastery Persnal_Growth
  /SCALE('ALL VARIABLES') ALL
  /MODEL=ALPHA
  /STATISTICS=DESCRIPTIVE SCALE
  /SUMMARY=TOTAL.
```

Reliability analysis

Notes

Output Created		23-JUN-2022 13:44:18
Comments		
Input	Data	/Users/macbookpro/Documents/Data(Spritual).sav
	Active Dataset	DataSet1
	Filter	<none>
	Weight	<none>
	Split File	<none>
	N of Rows in Working Data File	873
	Matrix Input	

Missing Value Handling	Definition of Missing	User-defined missing values are treated as missing.
	Cases Used	Statistics are based on all cases with valid data for all variables in the procedure.
Syntax		RELIABILITY<br><br>/VARIABLES=Psy_welbeing Work_Spritlty Compassion Relat_with_work Spritual_Orient Organz_Value Alingnm_Person_val Job_Stress Envonm_Mastery Persnal_Growth<br>/SCALE('ALL VARIABLES') ALL<br>/MODEL=ALPHA<br><br>/STATISTICS=DESCRIPTIVE SCALE<br>/SUMMARY=TOTAL.
Resources	Processor Time	00:00:00.00
	Elapsed Time	00:00:00.00

Scale: ALL VARIABLES

Case Processing Summary

		N	%
Cases	Valid	873	100.0
	Excluded[a]	0	.0
	Total	873	100.0

a. Listwise deletion based on all variables in the procedure.

Reliability Statistics

Cronbach's Alpha	N of Items
.981	10

Item Statistics

	Mean	Std. Deviation	N
Psychological Wellbeing	3.7892	1.08324	873
Workplace Spirituality	3.7640	1.07909	873
Compassion	3.9290	1.10856	873
Relationship with others at work	3.8396	1.06096	873
Spiritual Orientation	3.7881	1.06648	873
Organizational values	3.8202	1.04857	873
Alignment of Personal values	3.9164	1.10508	873
Job Stress	3.8625	1.07169	873
Environmental Mastery	3.9278	1.10796	873
Personal Growth	3.9003	1.09331	873

Item-Total Statistics

	Scale Mean if Item Deleted	Scale Variance if Item Deleted	Corrected Item-Total Correlation	Cronbach's Alpha if Item Deleted
Psychological Wellbeing	34.7480	80.893	.922	.978
Workplace Spirituality	34.7732	81.694	.881	.980
Compassion	34.6082	80.745	.907	.979
Relationship with others at work	34.6976	81.321	.919	.978
Spiritual Orientation	34.7491	81.846	.884	.980
Organizational values	34.7171	81.845	.901	.979
Alignment of Personal values	34.6208	80.726	.911	.979
Job Stress	34.6747	81.353	.907	.979
Environmental Mastery	34.6094	80.986	.894	.979
Personal Growth	34.6369	80.615	.928	.978

Scale Statistics

Mean	Variance	Std. Deviation	N of Items
38.5372	100.038	10.00189	10

```
FACTOR
  /VARIABLES PW1 PW2 PW3 PW4 PW5 PW6 WS1 WS2 WS3 WS4 WS5 COM1 COM2 COM3 COM4 ROW1 ROW2 ROW3 ROW4
    SO1 SO2 SO3 SO4 OV1 OV2 OV3 OV4 APV1 APV2 APV3 APV4 JS1 JS2 JS3 JS4 EM1 EM2 EM3 EM4 PG1 PG2 PG3 PG4
    PG5
  /MISSING LISTWISE
  /ANALYSIS PW1 PW2 PW3 PW4 PW5 PW6 WS1 WS2 WS3 WS4 WS5 COM1 COM2 COM3 COM4 ROW1 ROW2 ROW3 ROW4 SO1
    SO2 SO3 SO4 OV1 OV2 OV3 OV4 APV1 APV2 APV3 APV4 JS1 JS2 JS3 JS4 EM1 EM2 EM3 EM4 PG1 PG2 PG3 PG4 PG5
  /PRINT INITIAL CORRELATION SIG DET KMO AIC EXTRACTION ROTATION
  /CRITERIA MINEIGEN(1) ITERATE(25)
```

```
/EXTRACTION PC
/CRITERIA ITERATE(25)
/ROTATION VARIMAX
/METHOD=CORRELATION.
```

Factor Analysis

Notes

Output Created		23-JUN-2022 13:46:28
Comments		
Input	Data	/Users/macbookpro/Documents/Data(Spritual).sav
	Active Dataset	DataSet1
	Filter	<none>
	Weight	<none>
	Split File	<none>
	N of Rows in Working Data File	873
Missing Value Handling	Definition of Missing	MISSING=EXCLUDE: User-defined missing values are treated as missing.
	Cases Used	LISTWISE: Statistics are based on cases with no missing values for any variable used.

Syntax	FACTOR<br>/VARIABLES PW1 PW2 PW3 PW4 PW5 PW6 WS1 WS2 WS3 WS4 WS5 COM1 COM2 COM3 COM4 ROW1 ROW2 ROW3 ROW4<br>SO1 SO2 SO3 SO4 OV1 OV2 OV3 OV4 APV1 APV2 APV3 APV4 JS1 JS2 JS3 JS4 EM1 EM2 EM3 EM4 PG1 PG2 PG3 PG4<br>PG5<br>/MISSING LISTWISE<br>/ANALYSIS PW1 PW2 PW3 PW4 PW5 PW6 WS1 WS2 WS3 WS4 WS5 COM1 COM2 COM3 COM4 ROW1 ROW2 ROW3 ROW4 SO1<br>SO2 SO3 SO4 OV1 OV2 OV3 OV4 APV1 APV2 APV3 APV4 JS1 JS2 JS3 JS4 EM1 EM2 EM3 EM4 PG1 PG2 PG3 PG4 PG5<br>/PRINT INITIAL CORRELATION SIG DET KMO AIC EXTRACTION ROTATION<br>/CRITERIA MINEIGEN(1) ITERATE(25)<br>/EXTRACTION PC<br>/CRITERIA ITERATE(25)<br>/ROTATION VARIMAX

Resources	Processor Time	00:00:00.15
	Elapsed Time	00:00:00.00
	Maximum Memory Required	221088 (215.906K) bytes

Component Matrix[a]

	Component											
	1	2	3	4	5	6	7	8	9	10	11	12
PW1	.020	-.114	-.383	-.042	.302	.631	-.287	-.182	.257	.001	.202	-.062
PW2	-.010	-.082	-.378	-.007	.277	.619	-.223	-.163	.191	.023	.222	-.055
PW3	.019	-.115	-.395	-.026	.277	.651	-.281	-.160	.220	.039	.210	-.065
PW4	.043	-.339	.371	.277	.572	.024	.325	-.048	-.226	.104	.126	.040
PW5	.054	-.324	.361	.192	.567	.029	.326	-.025	-.267	.025	.194	.021
PW6	.064	-.352	.351	.257	.593	.031	.300	-.038	-.261	.099	.151	-.006
WS1	.047	.479	.199	-.075	-.068	.220	.583	-.327	.256	-.121	-.064	.010
WS2	.047	.470	.095	-.023	-.017	.258	.570	-.333	.249	-.125	-.055	-.023
WS3	.053	.493	.162	-.122	-.085	.229	.577	-.332	.279	-.133	-.075	-.033
WS4	.015	-.124	-.014	.086	.179	.134	.031	.093	.084	-.168	-.398	.520
WS5	.033	-.130	.002	.020	.185	.123	.004	.038	.063	-.118	-.432	.494
COM1	.941	.002	.035	.003	-.011	-.002	-.053	.007	.002	.006	.008	-.032
COM2	.082	.337	-.434	.299	.212	-.011	.251	.549	.083	.108	-.041	-.025
COM3	.050	.305	-.416	.284	.210	.028	.298	.544	.090	.031	.010	.005
COM4	.081	.327	-.439	.324	.180	-.024	.302	.546	.063	.076	-.006	.005
ROW1	-.013	-.049	.176	-.705	.306	-.124	.023	.320	.336	.120	.019	-.039
ROW2	.006	-.113	.169	-.685	.289	-.073	.103	.311	.310	.112	.027	-.062
ROW3	-.009	-.086	.212	-.720	.298	-.102	.056	.306	.353	.054	.001	-.039
ROW4	.146	.026	-.006	-.036	.072	.013	.011	-.003	.064	.012	.157	-.071
SO1	-.041	.146	-.054	.029	-.148	-.131	.093	-.048	-.011	.558	.167	-.347
SO2	-.020	.120	-.014	.029	-.186	-.118	.103	-.036	-.031	.541	.179	-.378

SO3	.922	.001	.046	.007	-.009	-.028	-.061	-.019	.014	-.012	.025	-.001
SO4	-.047	.103	-.077	.047	-.042	-.140	.029	.268	-.058	-.569	.423	-.059
OV1	-.021	.103	-.067	.024	-.045	-.146	-.026	.314	-.035	-.534	.415	-.020
OV2	.919	.012	.049	-.012	-.038	.001	-.035	.026	-.006	.020	-.008	-.030
OV3	-.069	.255	.376	.227	.151	.197	-.048	.027	-.059	.002	.117	-.108
OV4	-.076	.301	.380	.207	.163	.166	-.090	-.003	-.037	.079	.116	-.129
APV1	.925	-.002	.037	-.003	-.023	-.007	-.047	.011	.001	-.016	-.008	-.034
APV2	-.058	.296	-.064	.166	.444	-.518	-.293	-.311	.320	.013	-.001	.016
APV3	-.009	.293	-.080	.231	.379	-.501	-.250	-.321	.335	-.005	.013	.069
APV4	-.009	.344	-.102	.149	.423	-.541	-.290	-.325	.261	-.031	.046	.065
JS1	-.044	-.442	.217	.386	-.280	-.048	.066	.088	.606	-.043	.119	-.036
JS2	-.043	-.434	.227	.381	-.243	-.057	.032	.082	.579	-.046	.075	-.073
JS3	-.048	-.469	.226	.390	-.233	-.003	.105	.118	.611	-.033	.075	-.029
JS4	.939	.000	.035	.005	-.039	-.004	-.041	-.018	-.007	-.009	.015	-.013
EM1	.056	.033	-.070	.102	-.128	.045	.012	.045	.099	.531	.015	.451
EM2	.063	.040	-.025	.116	-.131	.041	.021	.079	.108	.506	.061	.466
EM3	.941	.025	.031	.000	.006	.002	-.049	.021	.042	-.019	.021	-.011
EM4	.021	.092	.011	-.181	-.201	-.088	.061	-.095	-.038	.037	.607	.496
PG1	.015	.125	.015	-.190	-.155	-.073	.131	-.102	-.058	.037	.620	.515
PG2	.904	.003	.023	.024	-.023	-.006	-.047	-.011	.017	-.041	-.033	-.004
PG3	-.106	.434	.514	.102	-.038	.252	-.393	.271	.014	.006	.004	.066
PG4	-.057	.421	.529	.111	-.024	.235	-.377	.283	.012	.031	-.009	.028
PG5	-.090	.427	.545	.113	-.013	.247	-.406	.242	.038	.042	-.012	.056

Rotated Component Matrix[a]

	Component											
	1	2	3	4	5	6	7	8	9	10	11	12
PW1	.015	.019	-.015	-.027	.014	.000	-.024	-.006	.935	-.023	-.020	-.033
PW2	-.022	-.029	.007	.008	-.021	-.029	-.020	.037	.879	-.021	.001	-.031
PW3	.011	-.008	-.027	-.016	-.025	-.017	-.009	.006	.937	-.014	-.031	-.004
PW4	.013	-.005	.000	.927	.011	.054	-.019	-.012	-.022	-.033	-.036	.003

PW5	.024	.039	-.007	.901	-.029	.004	-.035	-.013	-.018	.010	.029	-.059			
PW6	.034	.000	-.031	.948	.002	.023	-.009	-.010	.007	-.025	-.048	.002			
WS1	.021	.011	.928	-.002	-.004	-.015	.037	.002	-.049	-.011	.044	.022			
WS2	.015	-.039	.906	.012	.016	-.033	-.002	.064	.041	-.004	.005	.012			
WS3	.026	.037	.947	-.049	-.008	-.029	.025	.003	-.026	-.016	.018	.006			
WS4	.001	.001	-.012	.028	-.013	.041	-.024	.050	.026	.008	-.002	-.072			
WS5	.019	.022	-.002	.048	.005	-.009	.017	-.021	.035	-.032	-.027	-.001			
COM1	.943	.000	-.002	.019	-.010	-.015	-.011	.019	.006	-.020	.001	.002			
COM2	.039	-.014	.006	-.023	.045	-.041	.011	.926	.016	-.004	-.046	.019			
COM3	.006	-.007	.043	.006	.002	-.013	-.008	.903	.038	.057	.001	-.022			
COM4	.036	-.048	.020	-.019	.019	-.025	-.037	.929	-.013	.045	.003	.037			
ROW1	-.007	.931	-.020	-.009	.032	-.037	.034	-.017	-.005	.006	.021	.043			
ROW2	.005	.904	.006	.037	-.051	-.010	-.031	-.004	.003	-.023	.013	-.009			
ROW3	-.004	.947	.020	.004	-.001	-.008	.009	-.051	-.017	.013	-.009	-.066			
ROW4	.148	.081	.013	.024	.038	.023	-.037	.049	.086	.001	.102	.026			
SO1	-.029	-.011	.020	-.031	.032	-.018	-.012	.028	-.022	-.021	.013	.940			
SO2	-.005	-.018	.018	-.021	-.020	.005	.000	.004	-.043	-.008	.009	.943			
SO3	.925	-.004	-.001	.018	.023	-.005	-.022	-.009	-.005	-.002	.024	-.015			
SO4	-.031	-.022	.006	-.012	.002	-.005	-.013	.049	-.030	.951	.012	-.018			
OV1	-.003	.018	-.037	-.034	.010	-.003	.037	.048	-.027	.950	.017	-.011			
OV2	.921	.010	.007	.004	-.040	-.019	-.002	.024	-.022	-.033	.002	.007			
OV3	-.032	-.042	.053	.068	-.008	.001	.129	.000	-.006	.035	-.014	.001			
OV4	-.038	-.020	.049	.048	.054	-.021	.160	-.002	-.007	-.042	-.013	.007			
APV1	.927	.000	.006	.013	-.017	-.013	-.009	.010	-.007	-.003	-.016	-.007			
APV2	-.039	.032	-.015	.003	.931	-.016	.013	.013	-.002	-.018	-.041	.011			
APV3	.006	-.044	.028	-.001	.907	.033	.008	.028	-.009	.010	-.013	.006			
APV4	.007	-.006	-.010	-.019	.936	-.082	.001	.024	-.019	.021	.042	-.006			
JS1	-.016	-.027	-.018	.012	-.016	.931	-.013	-.040	-.017	.032	.000	.009			
JS2	-.012	-.025	-.052	.010	.003	.904	.000	-.029	-.026	-.043	-.009	-.005			
JS3	-.025	.000	-.005	.058	-.050	.935	-.017	-.009	-.001	.004	-.041	-.017			
JS4	.940	-.023	.016	.012	-.018	-.016	-.024	-.009	-.002	.004	.013	.010			
EM1	.026	-.024	.000	-.026	-.002	.006	-.004	.024	.014	-.042	.002	.034			

EM2	.036	-.012	.001	-.009	-.011	.036	.021	.035	-.008	-.008	.038	.010
EM3	.943	.022	.020	.010	.012	-.001	-.001	.041	.020	.001	.015	-.026
EM4	.014	.002	.001	-.053	-.008	-.004	.007	-.034	-.027	.000	.949	.013
PG1	.001	.017	.062	-.003	-.011	-.049	-.016	-.015	-.028	.029	.946	.004
PG2	.905	-.028	.022	.000	.004	-.003	-.022	.005	-.003	.010	-.027	-.022
PG3	-.052	-.007	.022	-.032	-.002	-.017	.927	-.007	-.013	.023	.016	-.010
PG4	-.002	.009	.010	-.017	-.005	-.008	.897	.007	-.035	.003	-.011	.006
PG5	-.033	.007	.026	-.014	.030	-.004	.922	-.036	-.009	.000	-.021	-.007

Component Transformation Matrix

Component	1	2	3	4	5	6	7	8	9
1	.994	-.003	.032	.035	-.018	-.030	-.056	.047	.007
2	.008	-.082	.472	-.332	.306	-.441	.423	.317	-.103
3	.053	.188	.156	.369	-.083	.228	.544	-.439	-.394
4	.004	-.738	-.076	.255	.191	.405	.116	.318	-.026
5	-.027	.320	-.061	.618	.445	-.269	-.025	.215	.308
6	-.010	-.108	.255	.033	-.560	-.037	.265	-.002	.683
7	-.074	.066	.628	.345	-.304	.074	-.428	.310	-.286
8	.004	.352	-.373	-.042	-.360	.109	.299	.618	-.190
9	.018	.382	.298	-.286	.349	.684	.024	.090	.256
10	-.017	.117	-.157	.094	-.008	-.051	.035	.089	.026
11	.010	.021	-.081	.199	.024	.113	-.006	-.015	.270
12	-.035	-.061	-.019	.023	.063	-.059	.063	-.007	-.079
13	.016	-.007	.035	.030	.012	-.028	.046	-.153	.073
14	-.011	.033	.106	.062	.017	-.102	-.066	-.197	.019
15	.017	.077	-.107	-.219	-.049	-.002	-.390	-.036	-.051

Direct and indirect (Mediation) hypothesized relationship

```
/* PROCESS for SPSS 2.16.3 */.
/* Written by Andrew F. Hayes */.
/* www.afhayes.com */.
/* Copyright 2012-2016 */.
/* Online distribution other than through */.
/* www.afhayes.com or processmacro.org is not authorized */.
/* Please read the documentation */.
/* available in Appendix A of */.
/* Hayes (2013) prior to use */.
/* www.guilford.com/p/hayes3 */.
/* Documentation available in Appendix A of http://www.guilford.com/p/hayes3 */.
preserve.
set printback=off.
```

Matrix

Notes

Output Created		23-JUN-2022 13:48:34
Comments		
Input	Data	/Users/macbookpro/Documents/Data(Spritual).sav
	Active Dataset	DataSet1
	Filter	<none>
	Weight	<none>

	Split File	<none>
	N of Rows in Working Data File	873

Syntax	matrix.<br>get dat/file = */variables =<br>Psy_welbeing<br>Work_Spritlty Job_Stress<br>Envonm_Mastery<br>Persnal_Growth /names =<br>vnames/missing = 9999.<br>compute ninit = nrow(dat).<br>get dat/file = */variables =<br>Psy_welbeing<br>Work_Spritlty Job_Stress<br>Envonm_Mastery<br>Persnal_Growth /names =<br>vnames/missing = omit.<br>get tmp/file = */variables =<br>Psy_welbeing /names =<br>yname/missing = omit.<br>get tmp2/file = */variables =<br>Work_Spritlty /names =<br>xname/missing = omit.<br>get tmp/file = */variables =<br>Job_Stress<br>Envonm_Mastery<br>Persnal_Growth /names =<br>mnames/missing = omit.<br>get tmp/file = */variables =<br>w999999t z999999t<br>v999999t q999999t.<br>compute wname=tmp(1,1).<br>do if (wname = ' ').<br>compute wname = 'xxx'.<br>end if.<br>compute zname=tmp(1,2).<br>do if (zname = ' ').<br>compute zname = 'xxx'.<br>end if.

Resources	Processor Time	00:00:03.89
	Elapsed Time	00:00:04.00

```
Run MATRIX procedure:

************* PROCESS Procedure for SPSS Release 2.16.3 ******************

        Written by Andrew F. Hayes, Ph.D.      www.afhayes.com
   Documentation available in Hayes (2013). www.guilford.com/p/hayes3

**************************************************************************
Model = 4
   Y = Psy_welb
   X = Work_Spr
  M1 = Job_Stre
  M2 = Envonm_M
  M3 = Persnal_

Sample size
       873

**************************************************************************
Outcome: Job_Stre

Model Summary
         R       R-sq        MSE          F        df1        df2          p
     .8466      .7167      .3258  2202.9493     1.0000   871.0000      .0000

Model
              coeff         se          t          p       LLCI       ULCI
constant      .6980      .0701     9.9513      .0000      .5603      .8356
Work_Spr      .8407      .0179    46.9356      .0000      .8056      .8759
```

```
**************************************************************************
Outcome: Envonm_M

Model Summary
          R       R-sq        MSE          F        df1        df2          p
      .7953      .6325      .4517  1498.9548     1.0000   871.0000      .0000

Model
              coeff         se          t          p       LLCI       ULCI
constant      .8543      .0826    10.3443      .0000      .6922     1.0163
Work_Spr      .8166      .0211    38.7163      .0000      .7752      .8580

**************************************************************************
Outcome: Persnal_

Model Summary
          R       R-sq        MSE          F        df1        df2          p
      .8111      .6579      .4093  1675.3104     1.0000   871.0000      .0000

Model
              coeff         se          t          p       LLCI       ULCI
constant      .8070      .0786    10.2647      .0000      .6527      .9613
Work_Spr      .8218      .0201    40.9306      .0000      .7824      .8612

**************************************************************************
Outcome: Psy_welb

Model Summary
          R       R-sq        MSE          F        df1        df2          p
      .9302      .8652      .1589  1393.1112     4.0000   868.0000      .0000

Model
              coeff         se          t          p       LLCI       ULCI
constant      .0902      .0532     1.6949      .0905     -.0142      .1946
Job_Stre     -.0564      .0273    -2.0655      .0392     -.1100     -.0028
```

```
Envonm_M      .1642      .0267     6.1471      .0000      .1117      .2166
Persnal_      .6894      .0302    22.8119      .0000      .6301      .7487
Work_Spr      .1550      .0257     6.0211      .0000      .1045      .2055

******************** DIRECT AND INDIRECT EFFECTS *************************

Direct effect of X on Y
    Effect         SE          t          p       LLCI       ULCI
     .1550      .0257     6.0211      .0000      .1045      .2055

Indirect effect of X on Y
          Effect    Boot SE   BootLLCI   BootULCI
TOTAL       .6532      .0320      .5912      .7184
Job_Stre    -.0474      .0284     -.1035      .0081
Envonm_M     .1340      .0240      .0886      .1812
Persnal_     .5666      .0292      .5085      .6222

Normal theory tests for specific indirect effects
          Effect         se          Z          p
Job_Stre    -.0474      .0230    -2.0630      .0391
Envonm_M     .1340      .0221     6.0690      .0000
Persnal_     .5666      .0284    19.9216      .0000

******************** ANALYSIS NOTES AND WARNINGS *************************

Number of bootstrap samples for bias corrected bootstrap confidence intervals:
    5000

Level of confidence for all confidence intervals in output:
   95.00

------ END MATRIX -----



restore.
```

```
/* PROCESS for SPSS 2.16.3 */.
/* Written by Andrew F. Hayes */.
/* www.afhayes.com */.
/* Copyright 2012-2016 */.
/* Online distribution other than through */.
/* www.afhayes.com or processmacro.org is not authorized */.
/* Please read the documentation */.
/* available in Appendix A of */.
/* Hayes (2013) prior to use */.
/* www.guilford.com/p/hayes3 */.
/* Documentation available in Appendix A of http://www.guilford.com/p/hayes3 */.
preserve.
set printback=off.
```

Matrix

Notes

Output Created		23-JUN-2022 13:49:02
Comments		
Input	Active Dataset	DataSet1
	Filter	<none>
	Weight	<none>
	Split File	<none>
	N of Rows in Working Data File	873

Syntax	matrix.<br>get dat/file = */variables =<br>Psy_welbeing Compassion<br>Job_Stress<br>Envonm_Mastery<br>Persnal_Growth /names =<br>vnames/missing = 9999.<br>compute ninit = nrow(dat).<br>get dat/file = */variables =<br>Psy_welbeing Compassion<br>Job_Stress<br>Envonm_Mastery<br>Persnal_Growth /names =<br>vnames/missing = omit.<br>get tmp/file = */variables =<br>Psy_welbeing /names =<br>yname/missing = omit.<br>get tmp2/file = */variables =<br>Compassion /names =<br>xname/missing = omit.<br>get tmp/file = */variables =<br>Job_Stress<br>Envonm_Mastery<br>Persnal_Growth /names =<br>mnames/missing = omit.<br>get tmp/file = */variables =<br>w999999t z999999t<br>v999999t q999999t.<br>compute wname=tmp(1,1).<br>do if (wname = ' ').<br>compute wname = 'xxx'.<br>end if.<br>compute zname=tmp(1,2).<br>do if (zname = ' ').<br>compute zname = 'xxx'.<br>end if.

Resources	Processor Time	00:00:03.85
	Elapsed Time	00:00:03.00

```
Run MATRIX procedure:

************* PROCESS Procedure for SPSS Release 2.16.3 *****************

        Written by Andrew F. Hayes, Ph.D.      www.afhayes.com
   Documentation available in Hayes (2013). www.guilford.com/p/hayes3

**************************************************************************
Model = 4
   Y = Psy_welb
   X = Compassi
  M1 = Job_Stre
  M2 = Envonm_M
  M3 = Persnal_

Sample size
      873

**************************************************************************
Outcome: Job_Stre

Model Summary
        R      R-sq       MSE         F       df1       df2         p
     .8412     .7077     .3361 2108.4620    1.0000  871.0000     .0000

Model
              coeff        se         t         p      LLCI      ULCI
constant      .6673     .0723    9.2296     .0000     .5254     .8092
Compassi      .8133     .0177   45.9180     .0000     .7785     .8480
```

```
**************************************************************************
Outcome: Envonm_M

Model Summary
          R       R-sq        MSE          F        df1        df2          p
      .8940      .7993      .2467  3468.4874     1.0000   871.0000      .0000

Model
              coeff         se          t          p       LLCI       ULCI
constant      .4171      .0619     6.7342      .0000      .2955      .5387
Compassi      .8936      .0152    58.8939      .0000      .8638      .9233

**************************************************************************
Outcome: Persnal_

Model Summary
          R       R-sq        MSE          F        df1        df2          p
      .8883      .7891      .2524  3258.8209     1.0000   871.0000      .0000

Model
              coeff         se          t          p       LLCI       ULCI
constant      .4582      .0626     7.3134      .0000      .3352      .5811
Compassi      .8761      .0153    57.0861      .0000      .8460      .9062

**************************************************************************
Outcome: Psy_welb

Model Summary
          R       R-sq        MSE          F        df1        df2          p
      .9390      .8818      .1394  1618.2376     4.0000   868.0000      .0000

Model
              coeff         se          t          p       LLCI       ULCI
constant      .1009      .0496     2.0316      .0425      .0034      .1983
Job_Stre     -.0751      .0236    -3.1842      .0015     -.1214     -.0288
```

```
Envonm_M      .0271      .0279      .9725      .3311     -.0276      .0819
Persnal_      .5972      .0295    20.2449      .0000      .5393      .6551
Compassi      .3926      .0308    12.7549      .0000      .3322      .4531

******************** DIRECT AND INDIRECT EFFECTS *************************

Direct effect of X on Y
    Effect         SE          t          p       LLCI       ULCI
     .3926      .0308    12.7549      .0000      .3322      .4531

Indirect effect of X on Y
          Effect    Boot SE   BootLLCI   BootULCI
TOTAL       .4864      .0336      .4225      .5544
Job_Stre    -.0611      .0143     -.0907     -.0342
Envonm_M      .0243      .0317     -.0367      .0895
Persnal_      .5232      .0300      .4621      .5800

Normal theory tests for specific indirect effects
          Effect         se          Z          p
Job_Stre    -.0611      .0192    -3.1758      .0015
Envonm_M      .0243      .0249      .9723      .3309
Persnal_      .5232      .0274    19.0780      .0000

******************** ANALYSIS NOTES AND WARNINGS *************************

Number of bootstrap samples for bias corrected bootstrap confidence intervals:
    5000

Level of confidence for all confidence intervals in output:
   95.00

------ END MATRIX -----



restore.
```

```
/* PROCESS for SPSS 2.16.3 */.
/* Written by Andrew F. Hayes */.
/* www.afhayes.com */.
/* Copyright 2012-2016 */.
/* Online distribution other than through */.
/* www.afhayes.com or processmacro.org is not authorized */.
/* Please read the documentation */.
/* available in Appendix A of */.
/* Hayes (2013) prior to use */.
/* www.guilford.com/p/hayes3 */.
/* Documentation available in Appendix A of http://www.guilford.com/p/hayes3 */.
preserve.
set printback=off.
```

Matrix

Notes

Output Created		23-JUN-2022 13:49:25
Comments		
Input	Active Dataset	DataSet1
	Filter	<none>
	Weight	<none>
	Split File	<none>
	N of Rows in Working Data File	873

Syntax	matrix.<br>get dat/file = */variables =<br>Psy_welbeing<br>Relat_with_work<br>Job_Stress<br>Envonm_Mastery<br>Persnal_Growth /names =<br>vnames/missing = 9999.<br>compute ninit = nrow(dat).<br>get dat/file = */variables =<br>Psy_welbeing<br>Relat_with_work<br>Job_Stress<br>Envonm_Mastery<br>Persnal_Growth /names =<br>vnames/missing = omit.<br>get tmp/file = */variables =<br>Psy_welbeing /names =<br>yname/missing = omit.<br>get tmp2/file = */variables =<br>Relat_with_work /names =<br>xname/missing = omit.<br>get tmp/file = */variables =<br>Job_Stress<br>Envonm_Mastery<br>Persnal_Growth /names =<br>mnames/missing = omit.<br>get tmp/file = */variables =<br>w999999t z999999t<br>v999999t q999999t.<br>compute wname=tmp(1,1).<br>do if (wname = ' ').<br>compute wname = 'xxx'.<br>end if.<br>compute zname=tmp(1,2).<br>do if (zname = ' ').

Resources	Processor Time	00:00:03.82
	Elapsed Time	00:00:04.00

```
Run MATRIX procedure:

************* PROCESS Procedure for SPSS Release 2.16.3 ******************

        Written by Andrew F. Hayes, Ph.D.      www.afhayes.com
   Documentation available in Hayes (2013). www.guilford.com/p/hayes3

**************************************************************************
Model = 4
   Y = Psy_welb
   X = Relat_wi
  M1 = Job_Stre
  M2 = Envonm_M
  M3 = Persnal_

Sample size
      873

**************************************************************************
Outcome: Job_Stre

Model Summary
         R       R-sq        MSE          F        df1        df2          p
     .8974      .8053      .2238  3603.3643     1.0000   871.0000      .0000

Model
              coeff         se          t          p       LLCI       ULCI
constant      .3820      .0602     6.3503      .0000      .2639      .5000
Relat_wi      .9065      .0151    60.0280      .0000      .8768      .9361
```

```
**************************************************************************
Outcome: Envonm_M

Model Summary
          R       R-sq        MSE          F        df1        df2          p
      .8174      .6682      .4078  1754.0146     1.0000   871.0000      .0000

Model
              coeff         se          t          p       LLCI       ULCI
constant      .6501      .0812     8.0075      .0000      .4908      .8095
Relat_wi      .8536      .0204    41.8810      .0000      .8136      .8937

**************************************************************************
Outcome: Persnal_

Model Summary
          R       R-sq        MSE          F        df1        df2          p
      .8157      .6653      .4005  1731.6162     1.0000   871.0000      .0000

Model
              coeff         se          t          p       LLCI       ULCI
constant      .6729      .0805     8.3631      .0000      .5150      .8308
Relat_wi      .8406      .0202    41.6127      .0000      .8009      .8802

**************************************************************************
Outcome: Psy_welb

Model Summary
          R       R-sq        MSE          F        df1        df2          p
      .9406      .8848      .1358  1666.0855     4.0000   868.0000      .0000

Model
              coeff         se          t          p       LLCI       ULCI
constant      .0426      .0493      .8631      .3883     -.0542      .1393
Job_Stre     -.2429      .0293    -8.2803      .0000     -.3005     -.1853
```

```
Envonm_M     .0877     .0254    3.4546     .0006     .0379     .1376
Persnal_     .7176     .0277   25.8919     .0000     .6632     .7719
Relat_wi     .4015     .0292   13.7680     .0000     .3443     .4588

******************** DIRECT AND INDIRECT EFFECTS *************************

Direct effect of X on Y
    Effect        SE         t         p      LLCI      ULCI
     .4015     .0292   13.7680     .0000     .3443     .4588

Indirect effect of X on Y
          Effect   Boot SE  BootLLCI  BootULCI
TOTAL       .4578     .0416     .3768     .5396
Job_Stre   -.2202     .0453    -.3101    -.1358
Envonm_M     .0749     .0183     .0414     .1140
Persnal_     .6031     .0279     .5485     .6571

Normal theory tests for specific indirect effects
          Effect        se         Z         p
Job_Stre   -.2202     .0268   -8.2016     .0000
Envonm_M     .0749     .0218    3.4419     .0006
Persnal_     .6031     .0274   21.9792     .0000

******************** ANALYSIS NOTES AND WARNINGS *************************

Number of bootstrap samples for bias corrected bootstrap confidence intervals:
    5000

Level of confidence for all confidence intervals in output:
   95.00

------ END MATRIX -----


restore.
```

```
/* PROCESS for SPSS 2.16.3 */.
/* Written by Andrew F. Hayes */.
/* www.afhayes.com */.
/* Copyright 2012-2016 */.
/* Online distribution other than through */.
/* www.afhayes.com or processmacro.org is not authorized */.
/* Please read the documentation */.
/* available in Appendix A of */.
/* Hayes (2013) prior to use */.
/* www.guilford.com/p/hayes3 */.
/* Documentation available in Appendix A of http://www.guilford.com/p/hayes3 */.
preserve.
set printback=off.
```

Matrix

Notes

Output Created		23-JUN-2022 13:49:46
Comments		
Input	Active Dataset	DataSet1
	Filter	<none>
	Weight	<none>
	Split File	<none>
	N of Rows in Working Data File	873

Syntax	matrix.<br>get dat/file = */variables = Psy_welbeing Spritual_Orient Job_Stress Envonm_Mastery Persnal_Growth /names = vnames/missing = 9999.<br>compute ninit = nrow(dat).<br>get dat/file = */variables = Psy_welbeing Spritual_Orient Job_Stress Envonm_Mastery Persnal_Growth /names = vnames/missing = omit.<br>get tmp/file = */variables = Psy_welbeing /names = yname/missing = omit.<br>get tmp2/file = */variables = Spritual_Orient /names = xname/missing = omit.<br>get tmp/file = */variables = Job_Stress Envonm_Mastery Persnal_Growth /names = mnames/missing = omit.<br>get tmp/file = */variables = w999999t z999999t v999999t q999999t.<br>compute wname=tmp(1,1).<br>do if (wname = ' ').<br>compute wname = 'xxx'.<br>end if.<br>compute zname=tmp(1,2).<br>do if (zname = ' ').<br>compute zname = 'xxx'.<br>end if.

Resources	Processor Time	00:00:03.78
	Elapsed Time	00:00:04.00

```
Run MATRIX procedure:

************* PROCESS Procedure for SPSS Release 2.16.3 ******************

        Written by Andrew F. Hayes, Ph.D.      www.afhayes.com
   Documentation available in Hayes (2013). www.guilford.com/p/hayes3

**************************************************************************
Model = 4
   Y = Psy_welb
   X = Spritual
  M1 = Job_Stre
  M2 = Envonm_M
  M3 = Persnal_

Sample size
      873

**************************************************************************
Outcome: Job_Stre

Model Summary
         R       R-sq        MSE          F        df1        df2          p
     .9076      .8238      .2026  4071.5767     1.0000   871.0000      .0000

Model
              coeff         se          t          p       LLCI       ULCI
constant      .4076      .0562     7.2467      .0000      .2972      .5180
Spritual      .9121      .0143    63.8089      .0000      .8840      .9401
```

```
**************************************************************************
Outcome: Envonm_M

Model Summary
          R       R-sq        MSE          F        df1        df2          p
      .7693      .5918      .5017  1262.7377     1.0000   871.0000      .0000

Model
              coeff         se          t          p       LLCI       ULCI
constant      .9004      .0885    10.1731      .0000      .7267     1.0741
Spritual      .7992      .0225    35.5350      .0000      .7551      .8433

**************************************************************************
Outcome: Persnal_

Model Summary
          R       R-sq        MSE          F        df1        df2          p
      .8533      .7281      .3254  2332.0675     1.0000   871.0000      .0000

Model
              coeff         se          t          p       LLCI       ULCI
constant      .5868      .0713     8.2316      .0000      .4469      .7267
Spritual      .8747      .0181    48.2915      .0000      .8392      .9103

**************************************************************************
Outcome: Psy_welb

Model Summary
          R       R-sq        MSE          F        df1        df2          p
      .9274      .8601      .1649  1334.3556     4.0000   868.0000      .0000

Model
              coeff         se          t          p       LLCI       ULCI
constant      .1287      .0542     2.3757      .0177      .0224      .2351
Job_Stre      .0643      .0327     1.9672      .0495      .0001      .1285
```

```
Envonm_M      .1988      .0265     7.5039      .0000      .1468      .2509
Persnal_      .7333      .0326    22.5192      .0000      .6694      .7972
Spritual     -.0604      .0335    -1.8045      .0715     -.1262      .0053

******************* DIRECT AND INDIRECT EFFECTS *************************

Direct effect of X on Y
    Effect        SE         t         p      LLCI      ULCI
    -.0604     .0335   -1.8045     .0715    -.1262     .0053

Indirect effect of X on Y
          Effect   Boot SE  BootLLCI  BootULCI
TOTAL       .8590     .0306     .7998     .9211
Job_Stre     .0587     .0285     .0042     .1163
Envonm_M     .1589     .0237     .1141     .2066
Persnal_     .6414     .0296     .5853     .7021

Normal theory tests for specific indirect effects
          Effect        se         Z         p
Job_Stre     .0587     .0298    1.9661     .0493
Envonm_M     .1589     .0217    7.3392     .0000
Persnal_     .6414     .0314   20.4056     .0000

******************* ANALYSIS NOTES AND WARNINGS *************************

Number of bootstrap samples for bias corrected bootstrap confidence intervals:
    5000

Level of confidence for all confidence intervals in output:
   95.00

------ END MATRIX -----



restore.
```

```
/* PROCESS for SPSS 2.16.3 */.
/* Written by Andrew F. Hayes */.
/* www.afhayes.com */.
/* Copyright 2012-2016 */.
/* Online distribution other than through */.
/* www.afhayes.com or processmacro.org is not authorized */.
/* Please read the documentation */.
/* available in Appendix A of */.
/* Hayes (2013) prior to use */.
/* www.guilford.com/p/hayes3 */.
/* Documentation available in Appendix A of http://www.guilford.com/p/hayes3 */.
preserve.
set printback=off.
```

Matrix

Notes

Output Created		23-JUN-2022 13:50:08
Comments		
Input	Active Dataset	DataSet1
	Filter	<none>
	Weight	<none>
	Split File	<none>
	N of Rows in Working Data File	873

Syntax	matrix.<br>get dat/file = */variables = Psy_welbeing Organz_Value Job_Stress Envonm_Mastery Persnal_Growth /names = vnames/missing = 9999.<br>compute ninit = nrow(dat).<br>get dat/file = */variables = Psy_welbeing Organz_Value Job_Stress Envonm_Mastery Persnal_Growth /names = vnames/missing = omit.<br>get tmp/file = */variables = Psy_welbeing /names = yname/missing = omit.<br>get tmp2/file = */variables = Organz_Value /names = xname/missing = omit.<br>get tmp/file = */variables = Job_Stress Envonm_Mastery Persnal_Growth /names = mnames/missing = omit.<br>get tmp/file = */variables = w999999t z999999t v999999t q999999t.<br>compute wname=tmp(1,1).<br>do if (wname = ' ').<br>compute wname = 'xxx'.<br>end if.<br>compute zname=tmp(1,2).<br>do if (zname = ' ').<br>compute zname = 'xxx'.<br>end if.

Resources	Processor Time	00:00:03.58
	Elapsed Time	00:00:03.00

```
Run MATRIX procedure:

************* PROCESS Procedure for SPSS Release 2.16.3 ******************

        Written by Andrew F. Hayes, Ph.D.      www.afhayes.com
    Documentation available in Hayes (2013). www.guilford.com/p/hayes3

**************************************************************************
Model = 4
    Y = Psy_welb
    X = Organz_V
   M1 = Job_Stre
   M2 = Envonm_M
   M3 = Persnal_

Sample size
        873

**************************************************************************
Outcome: Job_Stre

Model Summary
          R       R-sq        MSE          F        df1        df2          p
      .8342      .6959      .3497  1992.8490     1.0000   871.0000      .0000

Model
              coeff         se          t          p       LLCI       ULCI
constant      .6056      .0757     8.0044      .0000      .4571      .7541
Organz_V      .8526      .0191    44.6413      .0000      .8151      .8901
```

```
**************************************************************************
Outcome: Envonm_M

Model Summary
          R       R-sq        MSE          F        df1        df2          p
      .8130      .6610      .4166  1698.6105     1.0000   871.0000      .0000

Model
             coeff         se          t          p       LLCI       ULCI
constant      .6460      .0826     7.8230      .0000      .4839      .8080
Organz_V      .8591      .0208    41.2142      .0000      .8182      .9000

**************************************************************************
Outcome: Persnal_

Model Summary
          R       R-sq        MSE          F        df1        df2          p
      .8336      .6949      .3651  1984.0898     1.0000   871.0000      .0000

Model
             coeff         se          t          p       LLCI       ULCI
constant      .5799      .0773     7.5018      .0000      .4282      .7316
Organz_V      .8692      .0195    44.5431      .0000      .8309      .9075

**************************************************************************
Outcome: Psy_welb

Model Summary
          R       R-sq        MSE          F        df1        df2          p
      .9347      .8737      .1489  1500.8872     4.0000   868.0000      .0000

Model
             coeff         se          t          p       LLCI       ULCI
constant      .0359      .0520      .6906      .4900     -.0661      .1380
Job_Stre     -.0780      .0253    -3.0865      .0021     -.1276     -.0284
```

```
Envonm_M     .1376     .0260    5.3006     .0000     .0867     .1886
Persnal_     .6502     .0297   21.8873     .0000     .5919     .7085
Organz_V     .2560     .0260    9.8378     .0000     .2050     .3071

******************** DIRECT AND INDIRECT EFFECTS *************************

Direct effect of X on Y
    Effect       SE         t         p      LLCI      ULCI
     .2560     .0260    9.8378     .0000     .2050     .3071

Indirect effect of X on Y
          Effect   Boot SE  BootLLCI  BootULCI
TOTAL      .6169     .0276     .5630     .6720
Job_Stre  -.0665     .0248    -.1150    -.0182
Envonm_M   .1182     .0220     .0770     .1609
Persnal_   .5651     .0280     .5103     .6180

Normal theory tests for specific indirect effects
          Effect        se         Z         p
Job_Stre  -.0665     .0216   -3.0784     .0021
Envonm_M   .1182     .0225    5.2557     .0000
Persnal_   .5651     .0288   19.6399     .0000

******************** ANALYSIS NOTES AND WARNINGS *************************

Number of bootstrap samples for bias corrected bootstrap confidence intervals:
    5000

Level of confidence for all confidence intervals in output:
   95.00

------ END MATRIX -----



restore.
```

```
/* PROCESS for SPSS 2.16.3 */.
/* Written by Andrew F. Hayes */.
/* www.afhayes.com */.
/* Copyright 2012-2016 */.
/* Online distribution other than through */.
/* www.afhayes.com or processmacro.org is not authorized */.
/* Please read the documentation */.
/* available in Appendix A of */.
/* Hayes (2013) prior to use */.
/* www.guilford.com/p/hayes3 */.
/* Documentation available in Appendix A of http://www.guilford.com/p/hayes3 */.
preserve.
set printback=off.
```

Matrix

Notes

Output Created		23-JUN-2022 13:50:48
Comments		
Input	Active Dataset	DataSet1
	Filter	<none>
	Weight	<none>
	Split File	<none>
	N of Rows in Working Data File	873

Syntax	matrix.<br>get dat/file = */variables = Psy_welbeing Alingnm_Person_val Job_Stress Envonm_Mastery Persnal_Growth /names = vnames/missing = 9999.<br>compute ninit = nrow(dat).<br>get dat/file = */variables = Psy_welbeing Alingnm_Person_val Job_Stress Envonm_Mastery Persnal_Growth /names = vnames/missing = omit.<br>get tmp/file = */variables = Psy_welbeing /names = yname/missing = omit.<br>get tmp2/file = */variables = Alingnm_Person_val /names = xname/missing = omit.<br>get tmp/file = */variables = Job_Stress Envonm_Mastery Persnal_Growth /names = mnames/missing = omit.<br>get tmp/file = */variables = w999999t z999999t v999999t q999999t.<br>compute wname=tmp(1,1).<br>do if (wname = ' ').<br>compute wname = 'xxx'.<br>end if.<br>compute zname=tmp(1,2).

Resources	Processor Time	00:00:03.53
	Elapsed Time	00:00:04.00

```
Run MATRIX procedure:

************* PROCESS Procedure for SPSS Release 2.16.3 ******************

        Written by Andrew F. Hayes, Ph.D.      www.afhayes.com
   Documentation available in Hayes (2013). www.guilford.com/p/hayes3

**************************************************************************
Model = 4
   Y = Psy_welb
   X = Alingnm_
  M1 = Job_Stre
  M2 = Envonm_M
  M3 = Persnal_

Sample size
       873

**************************************************************************
Outcome: Job_Stre

Model Summary
          R       R-sq        MSE          F        df1        df2          p
      .8037      .6459      .4072  1588.7019     1.0000   871.0000      .0000

Model
              coeff         se          t          p       LLCI       ULCI
constant      .8102      .0796    10.1822      .0000      .6540      .9663
Alingnm_      .7794      .0196    39.8585      .0000      .7410      .8178
```

```
**************************************************************************
Outcome: Envonm_M

Model Summary
          R       R-sq        MSE          F        df1        df2          p
      .8324      .6929      .3774  1965.1520     1.0000   871.0000      .0000

Model
              coeff         se          t          p       LLCI       ULCI
constant      .6593      .0766     8.6067      .0000      .5090      .8097
Alingnm_      .8346      .0188    44.3300      .0000      .7976      .8715

**************************************************************************
Outcome: Persnal_

Model Summary
          R       R-sq        MSE          F        df1        df2          p
      .8625      .7440      .3064  2530.8840     1.0000   871.0000      .0000

Model
              coeff         se          t          p       LLCI       ULCI
constant      .5583      .0690     8.0889      .0000      .4228      .6938
Alingnm_      .8533      .0170    50.3079      .0000      .8201      .8866

**************************************************************************
Outcome: Psy_welb

Model Summary
          R       R-sq        MSE          F        df1        df2          p
      .9499      .9024      .1151  2005.7357     4.0000   868.0000      .0000

Model
              coeff         se          t          p       LLCI       ULCI
constant      .0146      .0454      .3226      .7471     -.0745      .1038
Job_Stre     -.0734      .0208    -3.5224      .0005     -.1143     -.0325
```

```
Envonm_M      .0806      .0229     3.5130      .0005      .0356      .1257
Persnal_      .5233      .0273    19.1726      .0000      .4697      .5769
Alingnm_      .4342      .0223    19.5015      .0000      .3905      .4779

******************** DIRECT AND INDIRECT EFFECTS *************************

Direct effect of X on Y
    Effect        SE         t         p      LLCI      ULCI
     .4342     .0223   19.5015     .0000     .3905     .4779

Indirect effect of X on Y
           Effect   Boot SE  BootLLCI  BootULCI
TOTAL        .4566     .0224     .4165     .5050
Job_Stre    -.0572     .0178    -.0915    -.0220
Envonm_M     .0673     .0163     .0358     .1000
Persnal_     .4466     .0255     .3979     .4975

Normal theory tests for specific indirect effects
           Effect        se         Z         p
Job_Stre    -.0572     .0163   -3.5076     .0005
Envonm_M     .0673     .0192    3.5012     .0005
Persnal_     .4466     .0249   17.9126     .0000

******************** ANALYSIS NOTES AND WARNINGS *************************

Number of bootstrap samples for bias corrected bootstrap confidence intervals:
    5000

Level of confidence for all confidence intervals in output:
   95.00

------ END MATRIX -----


restore.
```
